# Evaluation of Recommended Water Sample Collection Methods and the Impact of Holding Time on *Legionella* Recovery and Variability from Healthcare Building Water Systems

**DOI:** 10.3390/microorganisms8111770

**Published:** 2020-11-11

**Authors:** Marisa B. Hirsh, Julianne L. Baron, Sue M. Mietzner, John D. Rihs, Mohamed H. Yassin, Janet E. Stout

**Affiliations:** 1 Special Pathogens Laboratory, Pittsburgh, PA 15219, USA; mhirsh@specialpathogenslab.com (M.B.H.); jbaron@specialpathogenslab.com (J.L.B.); smietzner@specialpathogenslab.com (S.M.M.); jrihs@specialpathogenslab.com (J.D.R.); 2 Department of Infection Control, UPMC Mercy Hospital, Pittsburgh, PA 15219, USA; yassinm@upmc.edu; 3 Department of Civil and Environmental Engineering, University of Pittsburgh, Pittsburgh, PA 15261, USA

**Keywords:** *Legionella*, sample collection, sample processing, environmental water samples, culture, hold time, shipping time, concentration, distal site positivity

## Abstract

Water safety and management programs (WSMP) utilize field measurements to evaluate control limits and monitor water quality parameters including *Legionella* presence. This monitoring is important to verify that the plan is being implemented properly. However, once it has been determined when and how to sample for *Legionella*, it is important to choose appropriate collection and processing methods. We sought to compare processing immediate and flushed samples, filtration of different volumes collected, and sample hold times. Hot water samples were collected immediately and after a 2-min flush. These samples were plated directly and after filtration of either 100 mL, 200 mL, or 1 L. Additionally, unflushed samples were collected and processed immediately and after 1, 24, and 48 h of hold time. We found that flushed samples had significant reductions in *Legionella* counts compared to immediate samples. Processing 100 mL of that immediate sample both directly and after filter concentration yielded the highest concentration and percent sample positivity, respectively. We also show that there was no difference in culture values from time 0 compared to hold times of 1 h and 24 h. At 48 h, there were slightly fewer *Legionella* recovered than at time 0. However, *Legionella* counts were so variable based on sampling location and date that this hold time effect was minimal. The interpretation of *Legionella* culture results depends on the sample collection and processing methods used, as these can have a huge impact on the success of sampling and the validation of control measures.

## 1. Introduction

*Legionella* is an opportunistic waterborne pathogen that thrives in warm water environments and typically infects immunocompromised individuals [1]. Environmental surveillance has been found to be critical for evaluating the risk of healthcare-acquired Legionnaires’ disease. Additionally, routine environmental monitoring can assist in the management of *Legionella* in building water systems and can help reduce the chance of nosocomial illness [2]. Development of a water management plan based on ASHRAE Standard 188 is currently the industry standard best practice. However, whether conducting regular testing for *Legionella* spp. as a validation method to ensure the plan is controlling risk is recommended or required depends on the organization and guideline or standard [3,4,5,6,7,8].

In addition to there being organizationally varied requirements for testing, there are also a variety of sample collection volumes, flushing strategies prior to sampling, and sample holding time requirements prior to laboratory processing recommended by different technical guidelines for *Legionella* recovery (Table 1) [6,9,10,11,12,13]. Additionally, different sample volumes, sampling approaches, and sample holding times have been reported in numerous experimental laboratory studies [14,15,16,17,18]. Once it is determined that testing is going to be performed, there is unfortunately a lack of consensus for sampling and sample handling protocols, which can create confusion and impact the *Legionella* culture results [9]. Different methods may also answer different questions about the building water system, including what would a person expect to encounter when turning on the faucet or shower for use versus what is the water quality downstream in the cold water distribution system or the hot water recirculating system?

It is important to understand how these variables will affect the downstream *Legionella* results. Assessing risk is dependent upon reliable and accurate test results whether interpreting concentration (colony forming units (CFU) recovered) or proportion of outlets positive [19]. Therefore, we evaluated the effect of sample volume processed and use of flushing prior to sample collection on the presence of *Legionella* and the percentage of positive samples based on these standard recommendations. In addition, we evaluated the variability of *Legionella* concentrations based on sample hold time and sample location over time.

## 2. Materials and Methods

### 2.1. Effects of Flushing and Volume Tested on Legionella Recovery

Water was collected from 16 sink faucets weekly for 15 weeks in two buildings. Building A had expected concentrations of *Legionella* at >50 average colony forming units per milliliter (CFU/mL), while Building B had expected concentrations of <50 CFU/mL. Two samples were taken from each sink once the hot water outlet was opened: a 300 mL sample collected immediately and a 1000 mL sample after a 2-min flush. All collection bottles had sodium thiosulfate tablets present for chlorine neutralization. After collection, samples were immediately transported to the lab in an insulated cooler.

*Legionella* culture was conducted using a modified ISO method [21] with culture media plates prepared in-house from dehydrated media noted below, and 0.1 mL aliquots of the immediate, unflushed and post-flush samples were directly plated on buffered charcoal yeast extract agar (BCYE) (Remel, Lenexa, KS, USA) and BCYE with dyes, glycine, vancomycin, and polymyxin B (DGVP) [22]. The immediate samples were divided into a 100 mL portion and 200 mL portion that were concentrated by filtration using a 0.2 µm pore sized polycarbonate membrane. The entire liter of the post-flush samples was concentrated using the same protocol. The membranes were transferred to 10 mL tubes of sterile deionized water and vortexed, and 0.1 mL aliquots were plated to BCYE and DGVP. This filter concentrated 1 L sample collected after a 2-min flush, and was considered the ‘’reference method’’ for analytical comparisons. All culture plates were incubated 7 days in a humidified incubator at 36.5 ± 1 °C. During the incubation period, plates were examined two times, initially at 3–4 days and finally at 7 days. Latex agglutination (Oxoid Limited, Basingstoke, UK) and direct fluorescent antibody (DFA) staining were used to identify representative isolates of recovered *Legionella*.

### 2.2. Effects of Sample Holding Time on Legionella Recovery

Water was collected from 12 sink faucets in a hospital administrative and outpatient building, twice a week over 6.5 weeks for 13 sampling dates, for a total of 156 samples. The hot water outlets were opened, and 500 mL of the immediate water flow was collected into sterile bottles containing sodium thiosulfate to neutralize any residual chlorine in the water. The samples were cultured using a modified ISO method [21] immediately on site (T0), then again at 1 h (T1), 24 h (T24), and 48 h (T48) from collection for a total of 624 cultures. Water samples were held at room temperature throughout the testing period. On site at T0, 0.1 mL aliquots of each sample were plated onto two selective agar media: DGVP and BCYE with colistin, cephalothin, vancomycin, and cycloheximide (CCVC) (Becton, Dickinson, and Company, Franklin Lakes, NJ, USA). Upon returning to the laboratory, the T0 agar plates were placed into a humidified incubator at 36.5 ± 1 °C. After holding times of 1 h, 24 h, and 48 h (T1, T24, and T48), each water sample was plated directly and after filter concentration (100 mL was concentrated as above) onto BCYE and to DGVP agar plates. If the initial culture plates were overgrown, the filter concentrates were re-plated after pre-treatment with acid (0.2 M KCl, pH 2.2 ± 1). All sets of cultures were incubated for 7 days and examined for *Legionella* as above.

### 2.3. Statistical Analyses

#### 2.3.1. Flushing and Volume Analysis

Regression analysis was used to examine the variability in CFU counts as a function of building, sample processing, and water collection method (flushing). Given CFU is a count variable and not normally distributed, generalized linear regression modeling was used to examine the variability in CFU counts. The appropriateness of Linear, Poisson, and Negative Binomial probability density functions were compared using fit statics (e.g., Log Likelihood, Akaike’s Information Criterion, and Bayesian Information Criteria), and the negative binomial models were found to provide the most appropriate fit. *Legionella* counts were capped at 1000 CFU/mL based on the presence of outliers in the data. Chi-Square Test and Fisher’s Exact test were used to compare the proportion of positive samples by building, processing method, and collection method.

#### 2.3.2. Sample Holding Time and Location Analysis

Regression analysis was used to examine the variability in CFU counts as a function of hold time and sink location. Given CFU is a count variable and not normally distributed, generalized linear regression modeling was used to examine the variability in CFU counts. The appropriateness of Linear, Poisson, and Negative Binomial probability density functions was assessed using fit statics (e.g., Log Likelihood, Akaike’s Information Criterion, and Bayesian Information Criteria), and the negative binomial models were found to provide the most appropriate fit.

## 3. Results

### 3.1. Effects of Flushing and Volume Tested on Legionella Recovery

**Figure 1:**
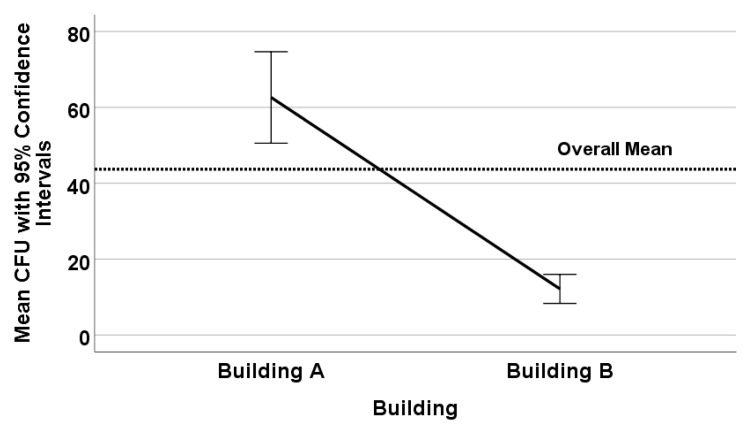
Mean *Legionella* counts (colony forming units per milliliter (CFU/mL)) as a function of building.

Over the course of this study, *Legionella pneumophila* serogroups 1, 4, and 5 were recovered and identified along with *Legionella* blue-white fluorescing species. A total of 240 water samples were collected and tested in this study: 150 from Building A and 90 from Building B. On average, the amount of *Legionella* recovered in all samples was 44 CFU/mL (±4.0 CFU/mL standard error). There was a statistically significant difference in counts from Building A (mean 63 CFU/mL ± 6.1 CFU/mL standard error) and Building B (mean 12 CFU/mL ± 1.9 CFU/mL standard error) (*p* < 0.0001) (Figure 1). On average, 75% of the samples were positive in Building A and 65% of the samples in Building B. There were significantly more positive samples per building than negatives (*p* < 0.001).

There was a statistically significant difference between the mean *Legionella* CFU/mL obtained from flushed and unflushed immediate samples, 23 CFU/mL vs. 57 CFU/mL, respectively (*p* < 0.0001). Flushed samples had an overall reduction in *Legionella* counts of 60% compared to unflushed samples. Additionally, there were significantly more positive unflushed samples on average (82%) than flushed (54%) (*p* < 0.001).

**Figure 2:**
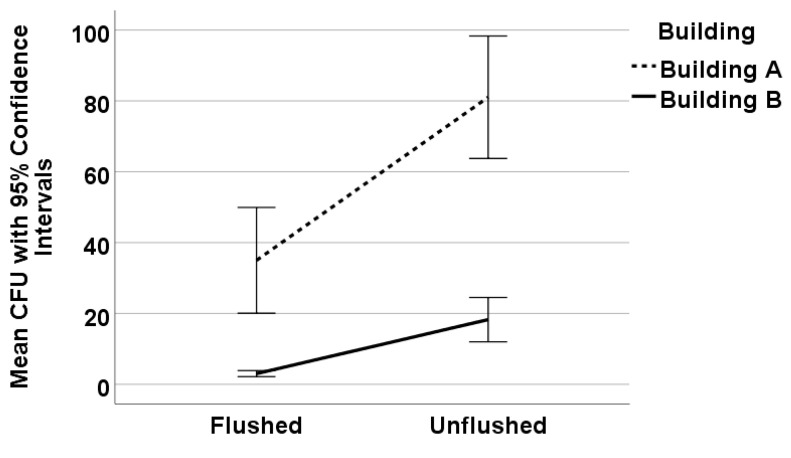
Mean *Legionella* counts (CFU/mL) as a function of flushing by building.

At the building level, the mean *Legionella* counts in Building A were significantly higher, 81 CFU/mL compared to 35 CFU/mL, in unflushed and flushed samples, respectively (*p* < 0.001) (Figure 2). There was overall a 63% reduction in *Legionella* concentrations based on flushing. In Building A, there was on average 60% positivity of flushed samples compared to 85% positivity in unflushed (*p* < 0.001).

In Building B, the average unflushed *Legionella* concentration (18 CFU/mL) was significantly higher than in flushed samples (3 CFU/mL) (*p* < 0.01) (Figure 2). Flushing resulted in an 87% reduction in *Legionella* species. On average, in Building B, 45% of flushed samples were positive, compared to 78% unflushed samples (*p* < 0.001).

Comparing all five processing methods demonstrated that the reference method, filter concentration of 1000 mL of the flushed sample, resulted in the lowest recovered *Legionella* concentration (Table 2). In general, the direct sample processing resulted in higher *Legionella* counts than the related filtered volume (*p* < 0.01) (Table 2). The other sample processing methods resulted in between 4.806 (filtered 200 mL unflushed) and 19.691 (direct unflushed) times higher *Legionella* concentrations than the reference method (Table 2). The percent positivity of samples ranged from 42% (direct flushed) to 90% (filtered 100 mL unflushed) (Table 2). Filtration of 100 mL or 200 mL of unflushed water samples resulted in statistically significant higher *Legionella* counts (*p* < 0.001) and positivity (*p* < 0.001) than filtration of 1000 mL of flushed water. There was a significant difference in the average CFU/mL between filtration of 100 mL and 200 mL (*p* < 0.001) but there was no significant difference between the percentage of positive samples (*p* = 0.567 Chi-Square test; *p* = 0.283 Fisher’s Exact test).

There were significant differences in the distribution of samples based on *Legionella* concentration category depending on the processing method (*p* < 0.001). Direct processed water samples were more likely to be non-detect for *Legionella* (28–54%) than any volume filtered (4–10%), whereas there were no low category direct processed samples. Additionally, direct processed water samples had more moderate concentrations of *Legionella* (22–33%) than the different volumes filtered (11–18%). High category concentration samples were not obtained using the reference method. These high category concentration samples were mostly observed in the direct unflushed samples (37%) (Table 3).

### 3.2. Effects of Sample Holding Time on Legionella Recovery

**Figure 3:**
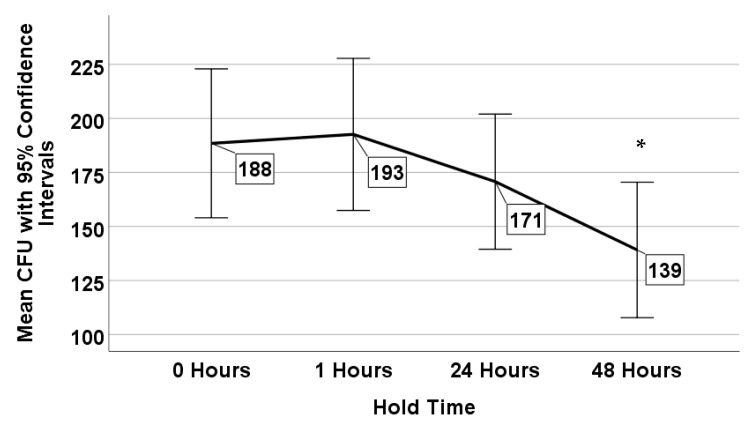
Mean *Legionella* counts (CFU/mL) as a function of hold time. * denotes a statistically significant difference (*p* < 0.05).

*L. pneumophila* was consistently recovered from 4 holding times of the 12 sinks throughout the 13 sampling dates. There was no significant difference in the colony counts between samples that were immediately processed (mean 188 CFU/mL ± 15.1 CFU/mL standard error) and those that were processed after 1 h (mean 193 CFU/mL ± 15.5 CFU/mL standard error) (*p* = 0.848) and 24 h of hold time (mean 171 CFU/mL ± 13.7 CFU/mL standard error) (*p* = 0.384) (Figure 3). However, there was a statistically significant reduction in colony counts after 48 h of holding time (mean 139 CFU/mL ± 11.2 CFU/mL standard error) (*p* = 0.008) compared to T0 (Figure 3).

**Figure 4:**
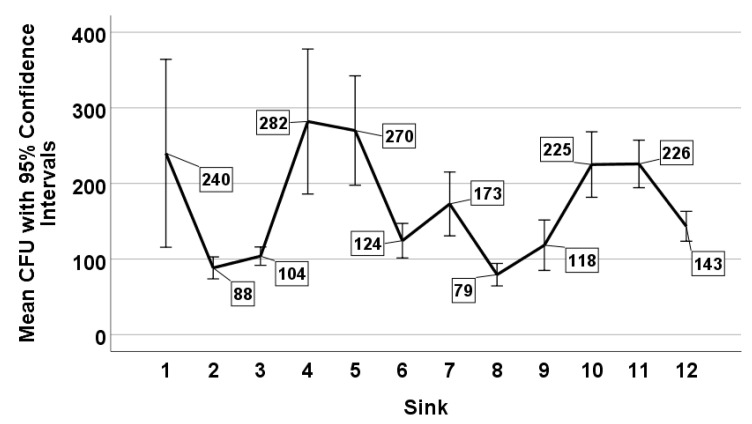
Mean *Legionella* counts (CFU/mL) as a function of sampling location.

*Legionella* concentrations were highly variable throughout the building, with the mean counts recovered ranging from 79 CFU/mL (sink 8) to 282 CFU/mL (sink 4) (Figure 4).

When comparing holding time and sink for their effect on the generalized linear model, sample location was found to account for more of the variability in *Legionella* concentrations than holding time (Table 4). To further analyze this finding and check for robustness, an ANOVA was also done. The effect size of holding time was not significant on *Legionella* counts (0.011 partial eta squared) (*p* = 0.073) but sink location was (0.111 partial eta squared) (*p* < 0.0001).

The average difference between the minimum and maximum mean recoveries for the 4 holding times was 54 CFU/mL (mean range 139–193 CFU/mL) (Figure 3), whereas the average difference based on the 12 sampling locations was 203 CFU/mL (mean range 79–282 CFU/mL) (Figure 4).

## 4. Discussion

Sample collection and processing are critical steps for environmental monitoring of *Legionella* in water systems [2,9]; however, a consensus regarding sample collection and processing approaches is still lacking across many technical guidelines [6,9,10,11,12,13,23]. Contradictions exist regarding whether to use first draw or post-flushing samples, various volumes of sample to filter concentrate, and how long the sample can be held after collection without compromising testing results. Additionally, thresholds or category levels of *Legionella* concentration have been proposed for specific action levels [6]. Few studies have been conducted to evaluate the effect of these variables on the *Legionella* results in terms of concentration or sample positivity.

Numerous previous studies suggest that the first draw sample without flushing maximizes the likelihood to recover *Legionella* from water samples and affects the concentration recovered [9,24,25,26]. The first 15 mL has been found to account for more than 50% of all the culturable bacteria found in the first liter [24]. This has been attributed to stagnation, which causes higher number of microorganisms due to microbial regrowth and disinfectant decay [9]. We also found that the *Legionella* counts were reduced, by 60% overall, after flushing the hot water for 2 min prior to sample collection. At the building level, this varied from a 63% to an 87% reduction in Building A versus Building B, respectively. Previous studies have seen similar reductions in the *Legionella* concentrations in hot water taps from sampling immediately and after a 3 min flush from 226 CFU/100 mL versus 45 CFU/100 mL (an 80% reduction) in the first liter and flushed samples, respectively [26]. Another study found a statistically significant reduction in total *L. pneumophila* concentration from 27,383 CFU/L vs. 19,462 CFU/L (a 28% reduction), including *L. pneumophila* serogroup 1 637 CFU/L versus 7 CFU/L (a 99% reduction), and *L. pneumophila* serogroups 2-14 26,746 CFU/L vs. 19,455 CFU/L (a 27% reduction) in the outlet versus a 2-min flush [25]. In the building with low expected counts (<50 CFU/mL), this could have a large impact on being able to recover any *Legionella* at all when collecting a flushed sample. If the building was the potential source of an outbreak and only flushed samples were collected, it may take multiple sampling attempts to recover an isolate to compare epidemiologically to a case of disease. As a public health measure, it may be useful for the public to flush water for several minutes prior to use for drinking or showering to reduce the load of *Legionella* present, especially in outlets with low or no prior use. Although this information is specific to *Legionella* species, this flushing may also provide benefit in reducing the concentration of other waterborne pathogens and potential exposure.

The overall sample positivity in the building was also found to be reduced by flushing, though not as much as the reduction in concentration. In Building A, positivity was reduced from 85% to 60% and in Building B from 78% to 45% after flushing. Positivity has been observed to be reduced after flushing previously. Samples positive for *Legionella* species were reduced from 35% positivity to 23%, comparing the hot water samples collected immediately and after a 3 min flush [26]. Cristina et al. noted a reduction in *L. pneumophila* serogroup 1 from 15.79% to 2.63% in hot water samples from the outlet versus 2-min flushed samples representing the plumbing system. Interestingly, this difference was not as pronounced for *L. pneumophila* serogroups 2-14, 42.11% outlet versus 40.79% flushed [25]. In our study, the low count building was again more affected by flushing than the higher count building, which can have implications for evaluation of risk. Collection of the first draw hot water also better represents a potential exposure event to an individual using that water source by capturing the initial slough off of bacteria from the biofilm in that specific outlet, which can allow for more targeted intervention if needed [27]. Sampling 15 mL instead of 1 L in first draw samples yielded 10 fold higher total bacterial counts, and after stagnation overnight, this was increased to 100 fold higher [24].

Directly plated cultures had the highest counts of *Legionella* in their collection method grouping (flushed versus unflushed) but the lowest sample positivity. This is likely due to a combination of effects including previously noted loss of recovery due to filter concentration [28]; that filter concentration allows for detection of low levels of *Legionella* in the samples. The ISO 11731:2017 [11] recommends multiple processing methods by sample type for the optimal recovery of *Legionella* in samples. The processing method also affected the category level that the *Legionella* concentration was found to be in. Most often, samples that were non-detect for *Legionella* were found in the direct plating whereas the low (<10 CFU/mL) concentration was only found in filtered samples. This evidence lends more support to the combination of effects noted and the advantage of using multiple processing methods. Processing an unflushed environmental water sample by both plating directly and after filter concentration of 100 mL yields both the best *Legionella* concentration and percent sample positivity. All sample positivity values exceeded 30%; however, the range within a collection method (42–67% flushed and 70–90% unflushed) varied by 20–25 percent. Depending on the collection and processing method selected, the *Legionella* concentration and positivity rate may artificially or incorrectly be found to be within regulatory limits. Interestingly, all concentrations of *Legionella* were significantly higher than the reference method (filtration of 1L after flushing), both in direct and filtered processing methods. No high concentration (≥100 CFU/mL) samples were recovered from this reference method. It has been noted that flushing reduces the probability of recovering *Legionella* if it is present in a sample [9,25,26], so this finding was expected but the magnitude (almost 20 times higher in directly plated, unflushed samples) was not. Although specific methods and studies have recommended larger sample volume for improving detection sensitivity, large sample volume often results in additional sampling costs, including supplies, labor, equipment, and shipment to the laboratory [9].

Previous studies evaluating effects of sample holding time on *Legionella* culture recovery observed competing results [17,18]. Holding water samples prior to processing was found to increase *Legionella* culture recovery [17]. However, a subsequent study observed that sample hold time had a small effect on *Legionella* culture results when compared to inherent methodology error, thus concluding that holding samples up to 48 h did not generate erroneous results [18]. The current study found there was no significant difference in *Legionella* culture results between time zero and up to 24 h of hold time. We found, however, a statistically significant reduction in *Legionella* counts after 48 h of holding time, not an increase. To reduce bias, we modeled from 12 sinks and 13 sampling dates to maximize the variability in these results. The *Legionella* concentration variability based on sampling location was significantly greater than the variability due to the hold time. The culture counts based on sampling location were so variable, they spanned 203 CFU/mL across the 12 sinks within the same building. For this reason, collection of multiple samples within the building is needed to allow for a more representative sampling. If too few samples are collected, a regulatory limit based on *Legionella* counts alone may be incorrectly met or missed. Previous studies have identified this variability in *Legionella* concentrations as a limitation of predicting disease risk based on counts alone. They have independently validated, based on environmental and clinical data, that when >30% of distal sites are positive for *Legionella* there is an increased risk of disease in individuals exposed to that water system [19,29,30,31,32,33].

Our results demonstrate that processing an immediate, unflushed 100 mL water sample produces the best possibility of recovering *Legionella* both based on count and sample positivity with direct and filter concentration processing, respectively. This sample processing can identify *Legionella* counts in all four defined category levels (non-detect, low, moderate, and high) [6]. Water samples processed within 24 h of collection show no change in *Legionella* counts due to hold time. As recommended by several organizations noted in Table 1, these samples should be received by the laboratory as soon after collection as possible for processing. While counts after 48 h were significantly less than in immediate processing, this change in concentration was substantially less than the variability of sampling locations in a building. To account for the extreme volatility in *Legionella* counts within a building, multiple sampling locations throughout the building should be analyzed and the percentage of positive distal sites should be considered in addition to the concentration. We recommend that in order to get a thorough picture of the *Legionella* risk in the building water system that immediate, unflushed hot water samples are collected from at least 10 distal outlets in the system that are then processed within 48 h of sample collection. Our results support educating the general public to allow water for drinking or showering to flush for several minutes, especially in outlets with suspected stagnation, to reduce the burden of waterborne organisms prior to use.

## Figures and Tables

**Table 1:** Summary of sample collection, holding time, and shipping recommendations.

	CDC [13]	ISO ^1^ [11]	AIHA [6]	ASTM [10]	ASHRAE [12]
Collection	Flush hot water for a few minutes until water is warm but not hot	No flush	Immediate and 2-min minimum flush	No flush	No flush to represent outlet conditions; flush to represent system water
Volume	1 L	1 L	125 mL–1 L for potable; 125 mL minimum for non-potable	10–100 mL for non-potable; >1 L for potable	250 mL for routine monitoring; 1 L for investigations
Holding Time or Shipping Recommendations	Not specified; Refrigerate if not processed within 72 h	Hold time 24 h–2 days; transport at 5 ± 3 °C is recommended, ambient temperature is acceptable	Ship overnight in an insulated container without ice	Ship overnight; Process within 48 h; Refrigerate if not processed in 72 h	Ship at ambient temperature within 24 h from collection; if time must exceed 48 h, consult with lab

CDC: Centers for Disease Control and Prevention; ISO: International Organization for Standardization; AIHA: American Industrial Hygiene Association; ASTM: American Society for Testing and Materials; ASHRAE: formerly the American Society of Heating, Refrigerating and Air-Conditioning Engineers. ^1^ The ISO 11731:2017 cites ISO 19458 “Water Quality—Sampling for Microbiological Analysis” for these recommendations [20].

**Table 2:** Descriptive statistics of *Legionella* based on processing and collection method.

Processing Method	Collection Method	Mean *Legionella* (CFU/mL)	Standard Error (CFU/mL)	Exp(B) ^1^	*p*-Value	Sample Positivity
Direct	Unflushed	106.08	15.6	19.691	<0.0001	70%
Direct	Flushed	40.67	9.4	7.548	<0.0001	42%
Filtered 100 mL	Unflushed	40.73	5.4	7.559	<0.0001	90%
Filtered 200 mL	Unflushed	25.67	2.9	4.806	<0.0001	88%
Filtered 1000 mL	Flushed	5.34	0.58	1	n/a	67%

n/a = Filtered 1000 mL was selected as the reference method. ^1^ Exp(B) is the exponential regression coefficient and represents the expected multiplicative effect on *Legionella* counts.

**Table 3:** Category level of *Legionella* concentration based on processing and collection method.

Processing Method	Collection Method	Non-Detect	Low (<10 CFU/mL)	Moderate (10–99 CFU/mL)	High (≥100 CFU/mL)
Direct	Unflushed	73 (28%)	0 (0%)	124 (33%)	43 (37%)
Direct	Flushed	139 (54%)	0 (0%)	82 (22%)	19 (16%)
Filtered 100 mL	Unflushed	25 (10%)	118 (26%)	69 (18%)	28 (24%)
Filtered 200 mL	Unflushed	11 (4%)	140 (31%)	62 (16%)	27 (23%)
Filtered 1000 mL	Flushed	10 (4%)	190 (43%)	40 (11%)	0 (0%)

**Table 4:** Omnibus test for model effects of sample holding time and sampling location.

Independent Variable	Wald Chi-Square	Degrees of Freedom	*p*-Value
Sink	114.660	11	<0.0001
Holding Time	11.055	3	0.011
